# Monitoring bioremediation of an impacted coastal lagoon through nature-based solutions: a case study of the Maricá Lagoon (RJ, Brazil)

**DOI:** 10.1007/s10661-026-15699-y

**Published:** 2026-07-24

**Authors:** Valquiria Maria de Carvalho Aguiar, Rodrigo Coutinho Abuchacra, Beatriz Freitas dos Santos Gonçalves, Joel de Mattos Junior, Joana Aiko Takiguchi Senra da Cruz, Paulo Henrique Campos, Milena Bertges de Carvalho Magalhães, Alcinei Rodrigues Machado, José Antônio Baptista Neto

**Affiliations:** 1 Programa de Pós-Graduação Em Geociências (Geoquímica), Departamento de Geoquímica, Instituto de Química, Universidade Federal Fluminense, Campus Do Valonguinho, Niterói, RJ Brazil; 2 Programa de Pós-Graduação Em Dinâmica Dos Oceanos E da Terra, Departamento de Geologia E Geofísica, Instituto de Geociências, Universidade Federal Fluminense, Av. General Milton Tavares de Souza, Niterói, RJ 24210-346 Brazil; 3 Programa de Pós-Graduação Em Geografia, Departamento de Geografia, Universidade Do Estado Do Rio de Janeiro (UERJ-FFP), Rua Dr. Francisco Portela, 1470, São Gonçalo, RJ 24435-005 Brazil

**Keywords:** Eutrophication, Effective microorganisms, Trophic state index, Hypoxia, Nutrients

## Abstract

This study evaluates bioremediation using effective microorganisms (EM) to improve quality in the eutrophic Maricá Lagoon (RJ, Brazil). The intervention, implemented in late 2021, consisted of the regular application of EM in one of the lagoon’s main fluvial contributors: the Mumbuca-Ubatiba drainage basin. To evaluate bioremediation, physicochemical and biogeochemical variables were monitored in the water column and sediments from 2020 to 2023. Whereas the drainage basin was sampled monthly, the lagoon was sampled every 3 months. Dissolved inorganic nitrogen (DIN) concentrations showed strong spatial variability in the system, reaching up to 2412 µM in the eastern margin (Mumbuca-Ubatiba)—dominated by ammonium (> 80% of DIN)—and up to 234 µM in the western margin (São Bento channel), where nitrate predominated (> 70%). Chlorophyll-a concentrations declined from 240 µg L⁻^1^ in 2020 to 11.44 µg L⁻^1^ in 2023 in Mumbuca-Ubatiba basin. Improvements associated with bioremediation were more evident in sedimentary variables in the lagoon. Total organic carbon decreased from 7.79% (2021) to 2.72% (2023), while total phosphorus showed an overall decreasing trend despite a peak of 2826 mg kg⁻^1^ in August 2023. The Trophic State Index (TSI) revealed a general improvement in water quality, with conditions shifting from hypereutrophic (TSI ≥ 59.1) and supereutrophic (58.2–59.0) to mesotrophic levels (53.2–55.7) in several stations. Despite improvements, persistent hypoxia (DO < 2 mg.L^−1^) and continuous high nutrient inputs indicate that bioremediation alone is insufficient to fully restore system conditions. These findings demonstrate that EM-based bioremediation should be integrated with broader environmental governance strategies for long-term effectiveness.

**Supplementary Information:**

The online version contains supplementary material available at 10.1007/s10661-026-15699-y.

## Introduction

Coastal lagoons are transitional ecosystems between continents and oceans, usually being characterized highly productive systems. Their specific features such as small depths, tidal effects, physicochemical gradients, and restricted circulation are the drivers of high production in these ecosystems. They have crucial ecological importance, acting as filters for marine and fluvial materials and being nurseries for several species, which makes coastal lagoons shelters of great biodiversity (Panigrahi et al., 2007; Pérez-Ruzafa et al., 2019a, 2019b). Lagoons provide multiple ecosystem services that include transport, recreation, and fisheries, among others (Newton et al., 2018).

The degradation of coastal environments driven by human activities causes heavy nutrient enrichment of aquatic systems and has greatly increased in the past decades, leading to eutrophication all around the world (Ferguson et al., 2024; Lenzi et al., 2013). Cultural eutrophication is one of the most common environmental issues affecting aquatic ecosystems worldwide. Accelerated urbanization, population growth, agricultural intensification, and inadequate wastewater management have gradually increased nitrogen and phosphorus loads into coastal waters, estuaries, and lagoons over recent decades (Akinnawo, 2023). The load of nutrients, i.e., nitrogen and phosphorus, originated from anthropogenic activities that reach coastal areas, has increased in the last decades, and eutrophication is still spreading to developing regions, despite the efforts of mitigation (Dai et al., 2023).

Habitat loss, hypoxia/anoxia of waters, dead zones in aquatic systems, decrease of biodiversity, and harmful algal blooms are among the main consequences of this process (Chandra et al., 2005).

The restricted circulation in coastal lagoons make them vulnerable to eutrophication and the accumulation of organic matter (Derolez et al., 2020; Pérez-Ruzafa et al., 2019a, 2019b), especially when accompanied by a high rate of urbanization in their surroundings which is the case of Maricá lagoon, in the state of Rio de Janeiro, Brazil. Over the past decades, this area has suffered the consequences of the economic growth of the region, including human occupation in its surroundings which turned it into a receptacle for high loads of macronutrients. Besides urbanization, the main anthropogenic activities in the surroundings of Maricá lagoon include livestock, primary sand extraction, and mechanical clay extraction (Amora-Nogueira et al., 2023). This increasing anthropogenic pressure is accompanied by the lack of public services, especially the ones regarding water distribution, sanitation system, and road infrastructure. Altogether, these activities are responsible for the increase of nutrients and suspended material driving eutrophication and silting of the Maricá lagoon. Local fauna and flora preservation is also under risk, with reduction of primary resources productivity, mainly fishing, due to the raw sewage that reaches the lagoon system (Laut et al., 2019; Toledo et al., 2021). Effects of eutrophication can be severe, and remediation of affected ecosystems has become an issue of essential importance for the preservation and recovery of urbanized coastal areas focusing not only on human health, but also on local economy (Malone & Newton, 2020).

The proposal of nature-based solutions has increased as a sustainable alternative for remediation of impacted aquatic systems. Bioremediation using effective microorganisms (EM) is a clean-up technology that accelerates natural degradation processes, relying on the ability of a consortium of microorganisms such as bacteria, algae, and fungi to transform organic matter, neutralizing pathogens and promoting biodiversity (Amorim et al., 2025; Ozkay et al., 2022). It is a nondestructive method that presents excellent cost-effectiveness. This technique was first developed in the 1970 s in Japan and has been applied worldwide since then (Velmurugan & Pandian, 2023; Zhao et al., 2013). The consortium of microorganisms is periodically released into the water bodies through solid (mud balls) or liquid matrices aiming to decrease excess organic matter, reduce macronutrient loads for algal growth, and improve sediments and water quality (Amorim et al., 2025). After their introduction in the aquatic system, effective microorganisms integrate the microbial food web and may interact with native microbial communities through competition, mutualistic associations, and nutrient cycling processes. These organisms can be consumed by protozoans, heterotrophic nanoflagellates, ciliates, meiofauna, and other bacterivorous organisms, contributing indirectly to higher trophic levels. However, the persistence of EM populations is generally considered limited because native microbial communities exert strong competition, environmental filtering, and predation, which interferes with the regulation of the abundance of introduced microorganisms over time (Safwat & Matta, 2021; Wakelin et al., 2008). Regarding potential ecological risks, the long-term effects of repeated EM applications in aquatic ecosystems remain insufficiently understood. Previous studies have reported improvements in water quality, organic matter degradation, and nutrient dynamics (Dondajewska et al., 2019; Zhao et al., 2013). A metagenomic study unveiled the increase of abundance and richness of microorganism in the substrate after bioremediation with EM of a channel in Maricá city (Pierri et al., 2023).

The bioremediation technique with EM has been carried out in the lagoon system of Maricá-Guarapina since November 2021, under the scope of a municipal program (“Lagoa Viva”) to increase the quality of the water bodies aiming benefits for the local population and economic development. The EM consortium applied in the lagoons is composed of bacilli (*Lactobacillus casei* and *Lactobacillus acidophilus*) and yeast (*Saccharomyces cerevisiae*) (Amorim et al., 2025) and is weekly applied to the lagoons and part of their drainage basin.

The effectiveness of EM in ecosystems exposed to strong spatial and seasonal variability under anthropogenic pressures remains largely unexplored. Although EM has been widely investigated in Europe and Asia (Dondajewska et al., 2019; Tang et al., 2025), existing studies have focused primarily on laboratory experiments and reservoir environments, limiting our understanding of its applicability to tropical ecosystems. The goal of this study is to evaluate the effectiveness of bioremediation with EM technique in the improvement of water and sediment quality of the Maricá lagoon, an ecosystem highly impacted by eutrophication, considering spatial and seasonal variability and persistent anthropogenic pressure.

## Materials and methods

### Study area

**Fig. 1:**
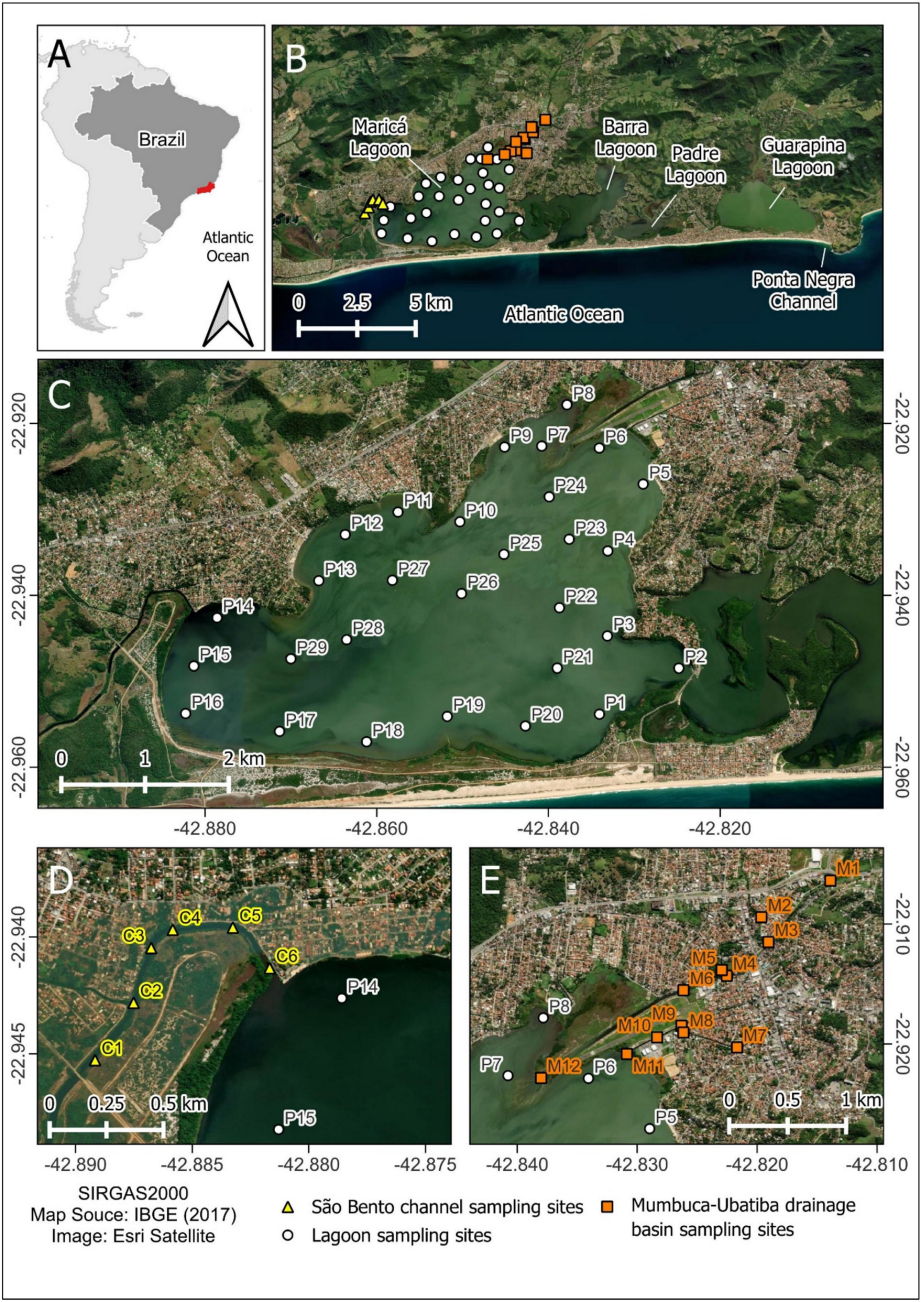
Study area within the Guarapina-Maricá lagoon system, located in the state of Rio de Janeiro, Brazil (**A**, **B**) and sampling sites in Maricá lagoon (**C**), São Bento channel (**D**), and the drainage basin of Mumbuca-Ubatiba (**E**)

The Maricá lagoon system is composed by four interconnected lagoons from east to west: Guarapina lagoon (~ 6.43 km^2^), Padre lagoon (~ 1.44 km^2^), Barra lagoon (~ 7.58 km^2^), and Maricá lagoon (~ 17.9 km^2^) itself (Laut et al., 2019) (Fig. 1). There is also the small Brava lagoon with and area of 1.2 km^2^, that discharges into Maricá lagoon, through the São Bento channel. The hydrographic basin of this lagoon system has approximately 330 km^2^ and a major portion is located in the city of Maricá (Laut et al., 2024). The Maricá lagoon water exchange with the sea occurs through Itaipuaçu channel; however, despite its long extension, it does not promote a good renewal. A better water renewal is promoted by Ponta Negra Channel at Guarapina lagoon (Laut et al., 2024).

Regarding environmental quality among the four lagoons, Maricá is the most impacted one, with several landfills surrounding it and discharge of raw sewage in its waters, an issue aggravated by its low depth. Massive fish kills are commonly observed in Maricá and Barra lagoons, probably caused by toxins produced by phytoplankton blooms, as well as anoxia/hypoxia of water column due to eutrophication (Amora-Nogueira et al., 2023; Pereira et al., 2025).

The lagoon system has experienced substantial land-use and land-cover changes over the last centuries, which have contributed to the progressive eutrophication of the area (Silvestre et al., 2017). The environmental history of the system can be divided into four major phases of anthropogenic intervention: (i) deforestation, the establishment of agricultural activities and fishing communities during the nineteenth century, followed by the opening of the Ponta Negra Channel in 1951, which connected the lagoon system to the sea; (ii) the construction of the Cordeirinho and Costa channels, establishing additional connections between Maricá Lagoon and the ocean on its eastern and western margins, respectively; (iii) improved regional accessibility resulting from the construction of roads and bridges; and (iv) increasing sewage discharge associated with urban expansion. Collectively, these interventions have caused significant environmental degradation, including reductions in lagoon water depth due to sediment accumulation and the intensification of eutrophication processes (Amora-Nogueira et al., 2023).

### Sampling

The sampling of the Mumbuca-Ubatiba drainage basin started in 2020. In the Maricá Lagoon and São Bento Channel, sampling began in 2021, with a three-month interval between each campaign. Altogether, ten campaigns have been made so far between January/2021 and August/2023 in Maricá Lagoon and São Bento Channel. The first two campaigns (January and August/2021) were made before the beginning of bioremediation with EM. The sampling area included 29 stations at Maricá lagoon (Fig. 1C), six stations at São Bento Channel (Fig. 1D), and 12 stations in the Mumbuca-Ubatiba drainage basin (Fig. 1E).

To consider seasonal variation, the sampling was divided into dry (May, August, and November) and wet seasons (January and February). Physicochemical variables, temperature, salinity, pH, dissolved oxygen (DO), and turbidity were measured with a multiprobe Horiba U50. The water column was sampled near the bottom with a Van Dorn bottle, and samples were immediately stored on ice. At Maricá lagoon, sediment sampling occurred with a stainless steel Van Veen grab equipped with a top opening, and samples were placed in plastic bags and immediately stored on ice. Regarding the bottom sediment, only the surface layer (up to 5 cm) was sampled, through the top opening.

### Laboratory analysis

In the laboratory, water samples were vacuum filtered with cellulose acetate membranes, with 0.45 µm porosity and filtrated water was used for determination of dissolved nutrients, total phosphorus, inorganic phosphorus, nitrate, nitrite, and ammonium. The membrane was used for determination of chlorophyll-*a* according to (Strickland & Parsons, 1972). The oxidation of samples for the determination of total dissolved phosphorus (TDP) was made with persulfate (APHA, 2005). Phosphate (PO_4_^3−^), nitrate (NO_3_^−^), nitrite (NO_2_^−^), and ammonium (NH_4_^+^) were determined through ion chromatography with a Metrohm IC Vario 940 equipped with conductivity and UV detectors. All reagents and stock standards solutions were analytical grade. Limits of detection (LOD) were defined as three times the signal-to-noise ratio (*S*/*N* = 3) and were 0.02–0.1 µM for NO₃⁻, 0.01–0.05 µM for NO₂⁻, 0.03–0.1 µM for PO₄^3^⁻, and 0.05–0.2 µM for NH₄⁺ under the analytical conditions used. Ultra-pure water Milli-Q (resistivity > 18.2 MΩ cm, 25 °C) was used for preparation of standards, dilutions, and cleaning of vials. Procedural blanks were included in each analytical batch and subjected to the same extraction and chromatographic procedures as the samples, ensuring control of background contamination and allowing verification that all reported concentrations were above blank levels. The sum of nitrite, nitrate, and ammonium forms was considered dissolved inorganic nitrogen (DIN).

Regarding phosphorus forms, dissolved inorganic phosphate (DIP) was determined in the stations of the drainage basin (Mumbuca-Ubatiba), whereas total dissolved phosphorus (TDP), regarding inorganic and organic phosphate was determined in the Maricá lagoon and São Bento Channel.

Sediment samples were freeze-dried and separated into aliquots for the determination of grain size, total organic carbon (TOC), total phosphorus, and calcium carbonate.

For the determination of grain size, organic matter was eliminated from the samples with H_2_O_2_ 10% (v/v) and heating. After that, carbonate was eliminated with HCl 10% (v/v) and heating. Grain size was then determined through laser scattering in a Malvern 2000 with a Hydro G unit. Data were processed with the software Gradistat® (Blott & Pye, 2001).

Total organic carbon was determined after elimination of carbonate with HCl 10% (v/v) through elemental analysis using a CHN/O using acetanilide as standard (Danovaro, 2009). The limit of detection (LOD) for TOC determination using the CHN elemental analyzer (PerkinElmer) was approximately 0.01 wt% C. Blanks were systematically included in each analytical batch under identical conditions to the samples, and their contribution was used for background correction when applicable.

Sedimentary total phosphorus (TP) was determined according to Aspila et al. (1976), based on acid extraction of ignited sediment samples followed by colorimetric determination of phosphate. Briefly, freeze-dried and homogenized sediments were ashed at 550 °C for 1 h to convert all phosphorus forms to orthophosphate, which was subsequently extracted using 1 M HCl. The resulting phosphate concentration in the extracts was quantified spectrophotometrically, and total phosphorus was calculated from calibration curves prepared with KH_₂_PO_₄_ standards. The limit of detection (LOD) for sedimentary total phosphorus determination, considering the analytical procedure and dilution steps, was estimated at 0.5 µg.P.g⁻^1^ for dry sediment.

### Data treatment

Statistical tests were performed using the software Statistica 10®. The nonparametric test of Mann–Whitney (*p* < 0.05) was used to evaluate the differences between wet and dry sampling periods, and Spearman analysis was used to perform correlation analysis.

Trophic index for Maricá lagoon was calculated based on the formula adapted by (Cunha et al., 2013) for tropical and subtropical areas. The index estimates the trophic level of the water body (Table 1) based on total phosphorus and chlorophyll-*a* concentration, according to the equations below:

$$TSI\, \left(Cla\right)=10x\left[6-\left(\frac{-\mathrm{0.2512}\,x\,\mathrm{ln}\,Cla+\mathrm{0.842257}}{\mathrm{ln}\,2}\right)\right]$$

$$TSI\, \left(TP\right)=10x\left[6-\left(\frac{-\mathrm{0.27637}\,x\,\mathrm{ln}\,TP+\mathrm{1.329766}}{\mathrm{ln}\,2}\right)\right]$$

$$TSI= \left(\frac{IET\left(Cla\right)+IET\left(TP\right)}{2}\right)$$

where Cla = concentration of chlorophyll-a in µg.L^−1^ and PT = total dissolved phosphorus in µg.L^−1^

**Table 1:** Classification of the trophic state according to TSI

Trophic state index	TSI
Ultraoligotrophic	TSI ≤ 51.1
Oligotrophic	51.2 ≤ TSI ≤ 53.1
Mesotrophic	53.2 ≤ TSI ≤ 55.7
Eutrophic	55.8 ≤ TSI ≤ 58.1
Supereutrophic	58.2 ≤ TSI ≤ 59
Hypereutrophic	TSI ≥ 59.1

## Results

**Fig. 2:**
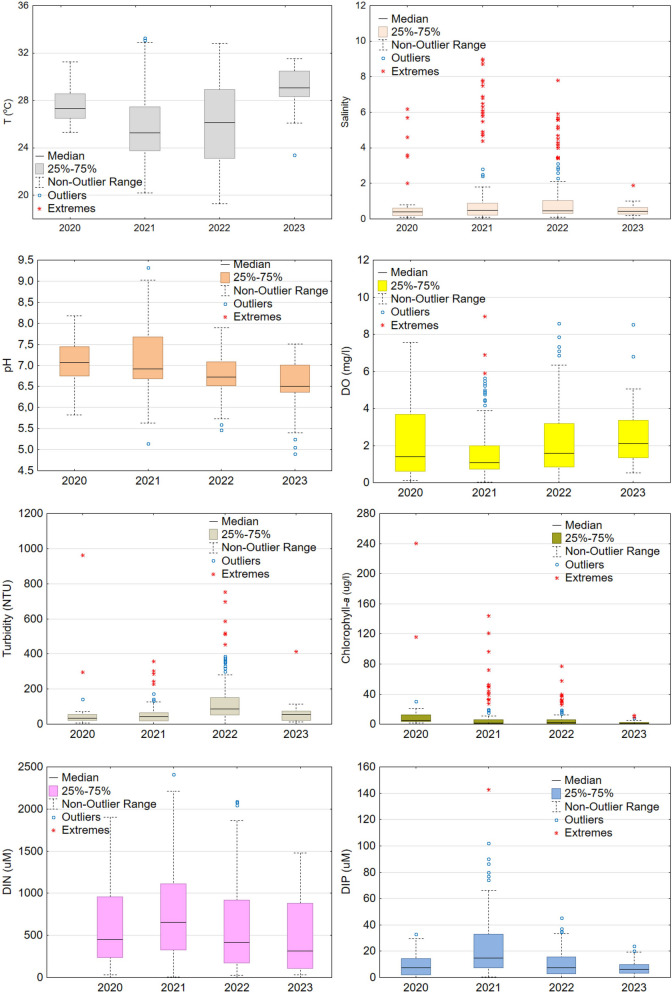
Annual variation for temperature, salinity, pH, dissolved oxygen (DO), turbidity, chlorophyll-*a*, dissolved inorganic nitrogen (DIN), and dissolved inorganic phosphorus (DIP) in Mumbuca-Ubatiba drainage basin

**Fig. 3:**
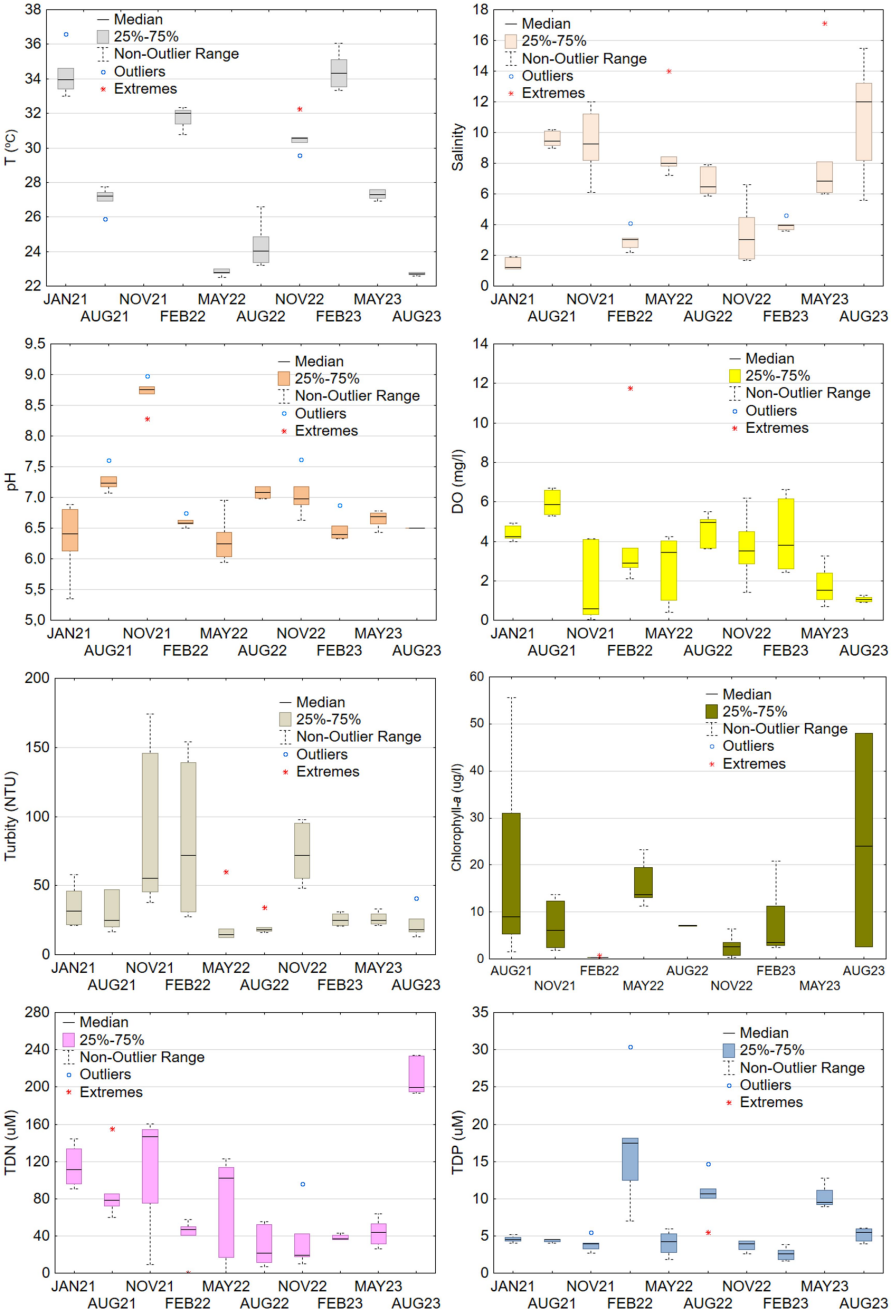
Annual variation for temperature, salinity, pH, dissolved oxygen (DO), turbidity, chlorophyll-*a*, total dissolved inorganic nitrogen (DIN), and total dissolved phosphorus (TDP) in the São Bento channel

**Fig. 4:**
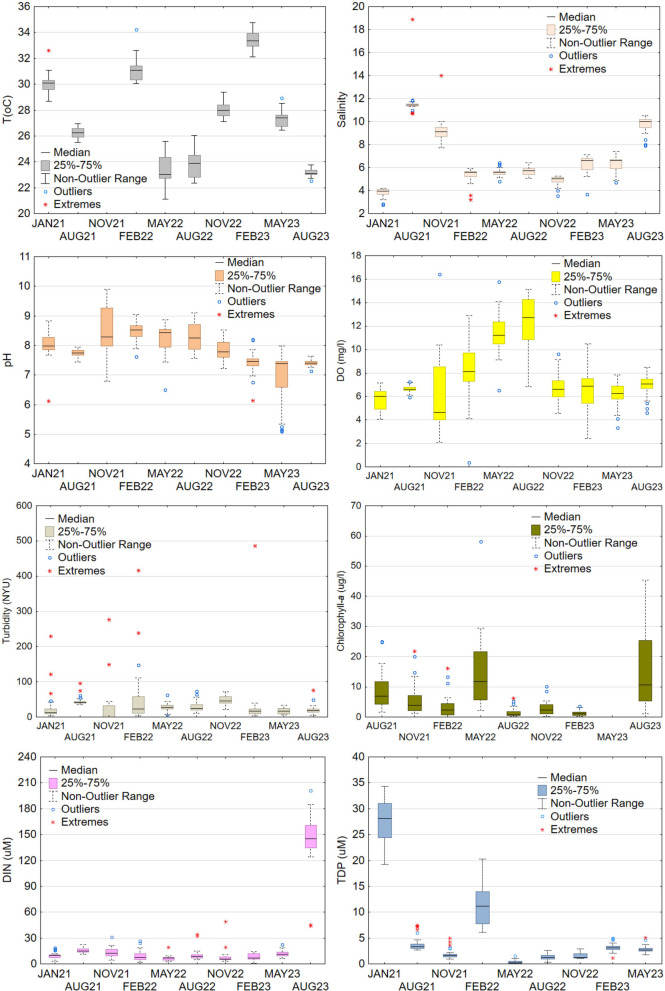
Annual variation for temperature, salinity, pH, dissolved oxygen (DO), turbidity, chlorophyll-*a*, dissolved inorganic nitrogen (DIN), and total dissolved phosphorus (TDP) in Maricá lagoon

The environmental monitoring of Mumbuca-Ubatiba drainage basin started in 2020, and the temporal series of the present study spans from 2020 to 2023; therefore, the basic statistics for this data set are presented on an annual basis (Fig. 2). For São Bento channel and Maricá Lagoon, the temporal series started in 2021, and samplings were made every three months; therefore, it was possible to present results for each campaign (Figs. 3 and 4). All detailed statistical results are provided in the Supplementary Material (Appendix A, Tables S1–S8).

### Mumbuca-Ubatiba drainage basin

The study area is subjected to strong seasonal variation between dry (autumn/winter) and wet (summer/spring) periods. The Mumbuca-Ubatiba drainage basin exhibited a marked seasonal variation that can be seen mainly through salinity, with significant differences between dry and wet seasons (*p* = 0.000004) and small effect size. The Mann–Whitney test revealed significant differences between dry and wet campaigns for most variables, except turbidity, nitrite, and nitrate (Tab. S1—Appendix A). Median values of pH were between 6.5 and 7.5. In 2023 the smallest values were registered and lower and upper quartiles for pH were, respectively, 4.83 and 6.58. The water in the drainage basin of Mumbuca-Ubatiba revealed concerning results pointing to hypoxia conditions, with median values of dissolved oxygen levels under 2.0 mg.L^−1^, in every year, except in 2023, when it was a little higher. Upper quartiles for DO were considered low for all the monitored years, between 2.00 (2021) and 3.67 mg.L^−1^ (2020). The lower quartiles pointed to hypoxia occurrence every year, varying from 0.62 (2020) to 1.34 mg.L^−1^ (2023). Turbidity was higher in 2020, reaching the extreme value of 962.00 NTU. The highest variability in turbidity was observed in 2022, with interquartile values of 51.80 and 150.00 NTU (Fig. 2). No significant differences were observed for turbidity between dry and wet seasons. The highest chlorophyll-*a* value, 240 µg.L^−1^, was registered in 2020, with lower and upper quartiles of 3.34 and 11.18 µg.L^−1^, respectively. Despite values of upper and lower quartiles, extreme chlorophyll-*a* values were registered every year, except in 2023, and differences between dry and wet seasons were considered significant (*p* = 0.012878) (Tab. S1 Appendix A). Most chlorophyll-*a* peaks were registered at stations M1, M2, M4, and M6, all of them located in the inner portion of the drainage basin (Fig. 1E). Despite the fact that nitrate and nitrite did not present significant seasonal difference (Tab. S1-Appendix A), DIN had the opposite result, due to extreme elevated concentrations of ammonium, which composed over 90% of DIN for the majority of samples in the four years of monitoring and presented significant difference between dry and wet seasons (*p* = 0.000081) (Table S1-Appendix A). Ammonium exhibited consistently high concentrations throughout the monitoring period, with maximum values observed in 2021 (1114.03 µM) and a general decrease towards 2023 (873.80 µM), while remaining the dominant fraction of dissolved inorganic nitrogen (> 90%). For nitrate, the upper quartile was 6.81 µM in 2020 and 8.71 µM in 2023, with the maximum upper quartile registered in 2021 (13.87 µM). Lower quartiles for nitrate were 0.07 µM in 2020 and 1.77 µM in 2023. Nitrite showed relatively stable distributions across years, with similar upper quartiles in 2020 and 2023 (3.51 and 3.82 µM, respectively) and low lower quartile values (0.09 and 0.28 µM, respectively). Extreme values of dissolved inorganic phosphorus were observed every year (Fig. 2). The highest variation occurred in 2021, with values of 7.54 and 32.97 µM for lower and upper quartiles, and a peak of 142.92 µM. Peaks of DIP were also registered in 2020 (33.12 µM), 2022 (45.21 µM), and 2023 (23.94 µM). Seasonal differences for DIP concentrations were considered significant (*p* = 0.000016) (Tab. S1-Appendix A).

### São Bento channel

The temporal dataset for São Bento Channel is from January 2021 to August 2023. Temperature and salinity variation over the monitored period revealed a strong seasonal variation between dry and wet periods (Fig. 3), with higher temperatures followed by lower salinity variation in the rainy period. Differences of temperature (*p* = 0.00000) and salinity (*p* = 0.00000) were considered statistically significant between dry and wet periods (Tab. S5- Appendix A). Upper quartiles of temperature varied from 31.70 to 33.90°C, whereas lower quartiles were between 23.00 and 26.92°C (Fig. 3). The upper quartiles for salinity were between 7.95 and 10.05 and lower quartiles varied from 2.46 to 3.95 (Fig. 3). Variation of pH did not present significant differences between dry and wet seasons, with upper quartiles varying between 6.93 and 7.24 and lower quartiles between 6.50 and 6.58. Dissolved oxygen did not present significant differences between dry and wet seasons and hypoxia was registered in the campaigns of November/21 (0.60–4.11 mg.L^−1^), May (0.40–4.23 mg.L^−1^) and November/22(1.43–6.2 mg.L^−1^), and May (0.7–2.39 mg.L^−1^) and August/23 (0.94–1.28 mg.L^−1^) (Fig. 3). Upper quartiles varied from 4.13 to 5.07 mg.L^−1^ whereas lower quartiles were between 1.10 and 2.87 mg.L^−1^. Turbidity varied significantly between wet and dry seasons (*p* = 0.000005) (Tab. S5-Appendix A), with upper quartiles between 43.05 and 58.90 NTU and lower quartiles varying from 18.65 and 21.35 NTU. The highest turbidity variations were registered in the wet season, November/21 (37.7–174 NTU) and February (27.7–154 NTU) and November/22 (48.2–97.6 NTU) (Fig. 3). Chlorophyll-*a* presented the highest variations in August/21 (1.60–55.54 µg.L^−1^) and August/23 (2.67–48.06 µg.L^−1^) (Fig. 3). The differences between dry and wet season were considered significant (*p* = 0.000299) for chlorophyll-*a* (Tab. S2-Appendix A). Chlorophyll-a exhibited relatively consistent distributions across the monitoring period, with values indicating strong episodic peaks in August/21 and August/23 and generally low background concentrations throughout the remaining campaigns. Total dissolved phosphorus presented significant differences (*p* = 0.004881; Tab. S5-Appendix A) between dry and wet seasons. Total dissolved phosphorus showed marked seasonal variability, with higher concentrations during wet-season campaigns and pronounced peaks in February/22 (7.05–30.41 µM), indicating episodic nutrient enrichment events (Fig. 3).

Regarding the DIN pool, nitrate had the higher contribution (between 70 and 98% of DIN) for most samples, except during the campaigns of May/22 and August/23 when ammonium was the dominant form (> 80% of DIN). Ammonium concentrations were generally low, except for episodic increases in 2020, followed by a marked reduction by 2023, when nitrate became the dominant DIN fraction in most samples. Nitrate remained the dominant inorganic nitrogen form throughout most of the monitoring period, with relatively stable distributions between 2020 and 2023. Nitrite consistently represented the lowest fraction of the DIN pool, with minor interannual variability. Total dissolved phosphorus presented significant differences between dry and wet seasons (*p* = 0.004875) (Tab. S2–Appendix A). The highest variation of TDP was observed in February/22 (7.05–30.41 µM). Overall, TDP exhibited significant seasonal variability, with higher concentrations during wet periods and episodic peaks, particularly in February/22, indicating seasonal driven nutrient inputs.

### Maricá Lagoon

The temporal dataset for Maricá lagoon is from January 2021 to August 2023. The marked seasonal variation of the study area was revealed in the form of temperature and salinity variations, with both variables presenting significant differences between dry and wet periods (Tab. S6-Appendix A). The upper quartiles for temperature varied between 29.71 and 32.95°C, and lower quartiles were between 23.35 and 26.26°C. Salinity presented the highest value, 18.90, in August 2021 (Fig. 4), and the upper quartiles varied between 5.70 and 11.38, whereas the lower quartiles were between 4.09 and 6.30. The variation of pH between dry and wet seasons was significant (*p* = 0.000023; Tab. S6; Appendix A) and upper quartiles varied between 7.49 and 8.46 (Fig. 4). Lower quartiles for pH were between 7.27 and 7.83. Dissolved oxygen presented significant differences between dry and wet seasons (*p* = 0.000000; Tab. S6; Appendix A), and the lowest concentrations were registered in the wet season in February/22 (0.37 mg.L^−1^-P12), characterizing hypoxia and in February/23 (2.40 mg.L^−1^-P19). Upper quartiles for DO in Maricá lagoon varied between 7.01 and 12.19 mg.L^−1^, whereas lower quartiles varied between 4.70 and 5.79 mg.L^−1^ (Fig. 4). Turbidity values did not vary significantly between wet and dry seasons, and upper quartiles were between 23.50 and 46.15 NTU; however, extreme values reached 486.00 NTU in February/23 (P7). Lower quartiles of turbidity values were between 5.09 and 20.80 NTU (Fig. 4). Chlorophyll-*a* concentrations were significantly different between dry and wet seasons, and upper quartiles were between 5.34 and 10.42 µg.L^−1^ (Fig. 4; Tab. S6-Appendix A); however, values as high as 58.21 µg.L^−1^ were registered in May/22 (P8). Lower quartiles for chlorophyll-*a* were between 0.80 and 2.49 µg.L^−1^. Regarding nitrogen forms, nitrite, nitrate, and ammonium presented significant differences between wet and dry seasons (Fig. 4) (Tab. S6-Appendix A). Upper quartiles for DIN varied between 3.49 and 10.0 µM, whereas lower quartiles varied from 0.43 and 1.81 µM. For most samples, the predominant nitrogen form in DIN was nitrate, except during November/22 and February/23 when ammonium was the dominant fraction. In August/23, the dominant nitrogen form in the DIN pool was nitrite. Nitrate showed interannual variability, with lower and upper quartiles of 6.29 and 12.25 µM in 2021 and 1.64 and 17.40 µM in 2023. For ammonium, the lower and upper quartiles were 0.34 and 3.49 µM in 2020 and 0.97 and 10.0 µM in 2023. Total dissolved phosphorus presented elevated values and variation in the initial campaign in January/21, with a maximum concentration of 34.29 µM (Fig. 4), and concentrations between dry and wet seasons varied significantly (Tab. S6-Appendix A). Upper quartiles for TDP varied between 3.35 and 24.42 µM, and lower quartiles were between 0.76 and 2.57 µM. There is no data of TDP for August/23, due to technical issues.

**Fig. 5:**
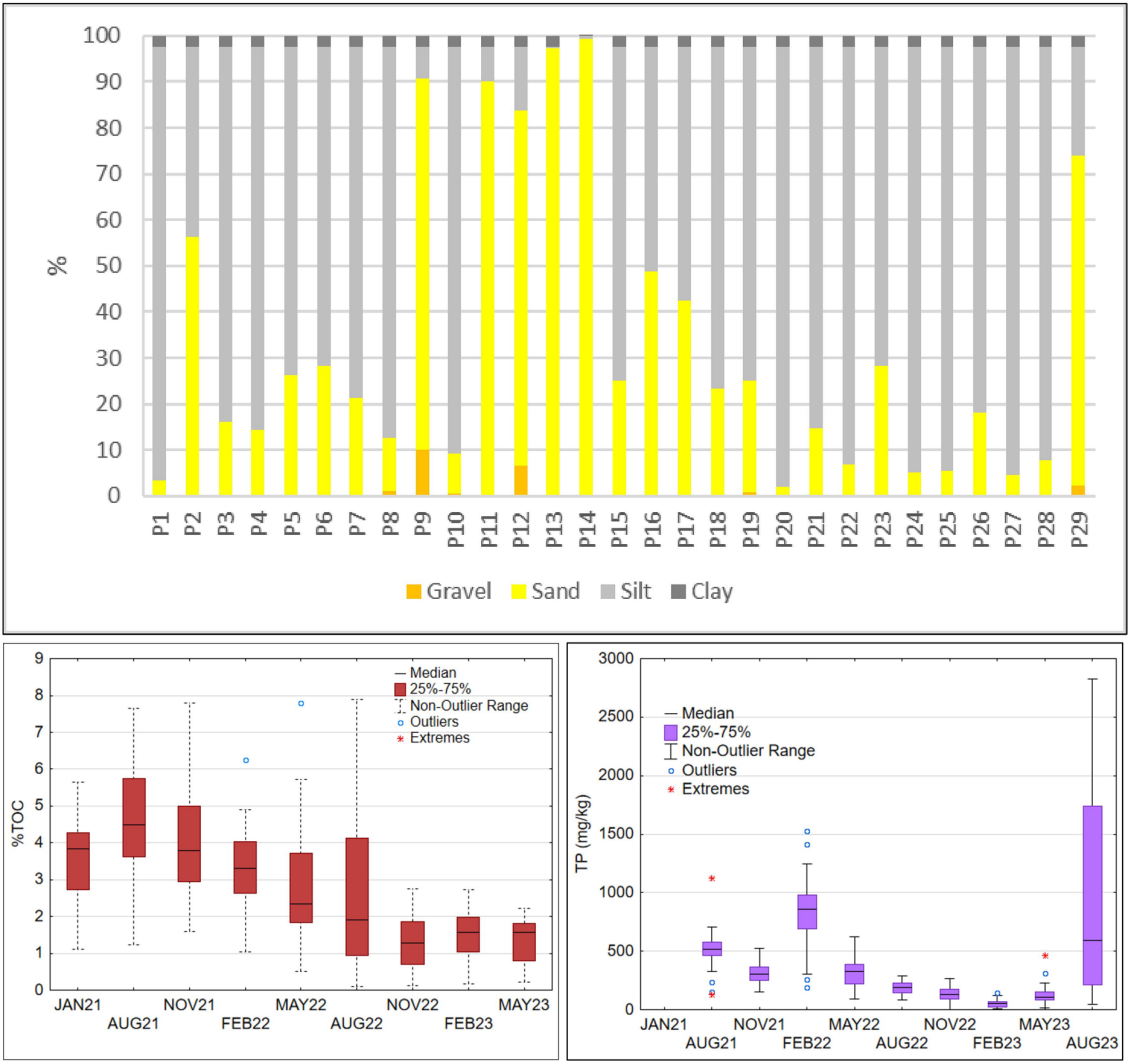
Grain size and annual variation for total organic carbon (TOC) and total phosphorus in (TP) in bottom sediments from Maricá lagoon

Regarding variables in bottom sediments of Maricá lagoon, grain size revealed the predominance of muddy sand in the bottom. The variation of total organic carbon was not considered statistically significant between dry and wet seasons (Tab. S6-Appendix A). Upper quartiles varied between 1.89 and 4.99% and lower quartiles were between 0.79 and 2.78% (Fig. 5); however, values as high as 7.79 (P18) and 7.89% (P1) were registered in May and August/22, respectively (Fig. 5). Sedimentary total phosphorus in bottom sediments varied significantly between wet and dry seasons (Tab. S6–Appendix A). Upper quartiles for TP varied between 469.89 (2021) and 261.68 mg.kg^−1^ (Fig. 5), and lower quartiles were between 3.30 and 59.70 mg.kg^−1^; however, in August/23, the concentrations of TP raised reaching values as high as 2826 mg.kg^−1^ (P28).

## Discussion

The effectiveness of effective microorganisms as a nature-based solution for recovery of natural environments regarding water and sediments has been increasingly studied in both natural environments (Amorim et al., 2025) regarding changes in organic matter degradation and in biodiversity, as well as in batch experiments concerning nutrient reduction and organic matter degradation (Lananan et al., 2014). A study comparing the use of EM alone, as well as in synergistic effect with microalgae (MA) on wastewater treatment (Lananan et al., 2014), has shown that the combination of both presents more effectiveness in bioremediation, since EM produce carbon dioxide and consume oxygen and MA phytoremediation has the opposite effect. Lananan et al. (2014) have demonstrated the decrease of oxygen levels to almost zero in the very first few days of a batch experiment to reduce nutrients of aquaculture wastewater using only EM. Dissolved oxygen plays a key role in regulating microbial processes involved in nutrient cycling, particularly nitrification and organic matter degradation, which can contribute to reductions in nutrient concentrations and, according to Ji et al. (2014), the abundance of DO in the environment, during bioremediation with EM, has proved to help decrease the concentrations of several substances in impacted water bodies, such as total phosphorus and ammonia. Lagoon systems are natural environments where oxygen levels are renewed naturally by microalgae and through mixing of the water column by winds, due to the low depth of its waters (Leoni et al., 2022). In this context, it was expected that the use of EM in an aerobic environment would help to increase the effectiveness of the bioremediation process, through a synergistic effect, generating conditions to improve the action of EM.

The renewal of waters in Maricá lagoon occurs mainly through the fluvial discharge in its waterbody. In the area under the influence of Mumbuca river mouth, the renewal of water is around 60%; however, new waters do not necessarily correspond to clean waters. Results revealed that Maricá lagoon was strongly impacted by anthropogenic nutrient input through the Mumbuca-Ubatiba drainage basin, and by the São Bento Channel, a tidal channel that receives freshwater discharge. Between the two main fluvial sources, Mumbuca river stood out as the main contributor of anthropogenic load to the lagoon, with turbid waters, loaded with dissolved nitrogen and phosphorus and accompanied by elevated phytoplankton biomass, characterizing a hypertrophic waterbody.

The evaluation of ecosystem health is a hard task and should not be addressed with a unique variable; however, dissolved oxygen is considered an important tool regarding this analysis (Cravo et al., 2020), since it is essential for the survival of heterotrophic organisms. A minimal DO concentration is required for an aquatic ecosystem to be considered healthy (Esteves 2011) since, below a certain level, adverse effects are usually expected with deleterious consequences over biological processes and subsequent economic effects (Banerjee et al., 2019). Even though different species might respond differently to changes in DO, concentrations under 5 mg.L^−1^ are considered stressful for biota, whereas values < 1–2 mg.L^−1^ create adverse effects for the growth of biota and threatens their survival (Rouf et al., 2022). The Mumbuca-Ubatiba drainage basin was characterized by an unbalanced range of oxygen levels, where periods with high concentrations of DO (up to 8.96 mg.L^−1^) alternated with hypoxia/anoxia events throughout the four years of monitoring. The median of DO during the first three years of monitoring, characterizing hypoxia, i.e., levels of oxygen below 2 mg.L^−1^ (Byers et al., 2023; Dugener et al., 2023), suggesting that this process is well established in the sampling area as a consequence of the permanent input of organic matter in the water courses of the basin. A small increase of oxygen levels was registered in 2023 (Fig. 2), with higher values of median and 25th percentile in the last year of monitoring; however, this feature was not considered statistically significant between pre and post bioremediation periods.

Concentrations of DIN in Mumbuba-Ubatiba drainage basin surpassed 2000 µM. A small decrease in DIN was observed between 2020 and 2023 (Fig. 2), with a lower median value, as well as a shorter range in the last year of monitoring. This decrease was considered significant for wet season measurements, when anthropogenic input is usually higher due to increased fluvial discharge. Considering that the highest contribution of nitrogen Mumbuca-Ubatiba was in the form of NH_4_^+^, which corresponded to over 80% of DIN in most of the samples. These patterns suggest a strong anthropogenic influence most likely associated with untreated sewage inputs, which are known to increase ammonium concentrations in aquatic systems (Cabral et al., 2019). Organic matter and urea are among the main components of raw sewage, and both can originate ammonium through hydrolyses and organic matter degradation, respectively. The mineralization of excess organic matter is usually responsible for oxygen depletion in the water column releasing ammonium (Kendall et al., 2014; Rana et al., 2019). The exhaustion of oxygen levels, however, inhibits nitrification (Jechalke et al., 2011), a hypothesis corroborated by low NO_3_^−^ values (< 2% of the DIN) for most of the samples in the Mumbuca-Ubatiba drainage basin, as well as the low concentrations of DO. However, the lack of sanitation and high degree of urbanization in the drainage basin reinforces the hypothesis of mineralization of organic matter as the main source of ammonium in the system.

The elevated concentrations of ammonium in the Mumbuca-Ubatiba drainage basin might be potentialized by bioremediation with EM since the decomposition of organic matter degradation releases inorganic nutrients. Components of organic matter include dissolved organic nitrogen, which is made of labile and recalcitrant forms, and low molecular weight nitrogen compounds are substrate to ammonification (Romillac, 2019). In terms of primary production, the use of NH_4_^+^ over NO_3_^−^ has a lower energetic cost in terms of the cells associated with protein synthesis, and some studies describe that the inhibition of nitrate by ammonium is highly variable (Dortch, 1990; Olofsson et al., 2019). On the other hand, primary production might be inhibited by NH_4_^+^ (Parker et al., 2012). Ammonium is also used by macrophytes, which are known for removing excess nutrients from waters (Srivastava et al., 2008), and the proliferation of macrophytes in areas impacted by large concentrations of nutrients is well documented (Clavier et al., 2005). The floating aquatic weeds observed in sampling points suggest the use of inorganic nutrients by macrophytes, a condition that characterizes eutrophication scenarios. Despite the potential of aquatic weeds in recycling inorganic nutrients with consequent lowering levels in the water column (Pinto-Coelho & Barcelos Greco, 1999), their proliferation constitutes a problem since these floating mats interfere with fishing, obstruct routes for navigation, and cause losses of water in irrigation systems (Gunnarsson & Petersen, 2007). In the Mumbuca-Ubatiba drainage basin, the floating macrophyte mats were frequently observed during the wet season, probably because of increased nutrient inputs transported by river discharge and surface runoff from the watershed. The enhanced nutrient availability, combined with higher temperatures and solar radiation, created favorable conditions for macrophyte proliferation.

This undesirable effect of macrophytes may be driven by the enhanced mineralization of organic matter induced by EM elevating ammonium concentrations in aquatic systems (Edwards et al., 2024), potentially favoring the growth of macrophytes under eutrophic conditions. In addition, dense macrophyte stands may reduce phytoplankton biomass through competition for nutrients and light (Wang et al., 2025). Although these mechanisms were not directly evaluated in the present study, the observed increase in macrophyte abundance and the concomitant decrease in chlorophyll-a concentrations might corroborate this hypothesis.

The decrease of DIP was observed from 2020 to 2023, despite the initial elevated concentrations in fluvial waters (up to 142.92 µM in 2021), reaching the maximum value of 23.94 µM in the past year (Fig. 2). In the same way of dissolved inorganic nitrogen, DIP is also released by the degradation of organic matter and can be recycled by the floating weeds especially in the rainy season, when this condition was frequently observed. Spearman analysis corroborated the hypothesis of large inputs or organic matter reaching the drainage basin and posteriorly suffering mineralization, as indicated by significant and inverse correlation between NH_4_^+^ and DO. Inverse and significant correlations between DO and DIP also corroborate the hypothesis of mineralization of organic matter, releasing inorganic forms of nutrients to the water column while consuming oxygen.

High turbidity of waters in the drainage basin, reaching values as high as 962 NTU, also suggested input of high loads of suspended material, probably in the form of raw sewage, and seasonal changes did not seem to affect this variable, pointing to a permanent input of anthropogenic loads in this water course. However, turbidity range suffered a noticeable decrease in the last year of monitoring (Fig. 2), which was accompanied by the decrease in phytoplankton bloom. Concentrations of phytoplankton biomass, expressed as chlorophyll-*a*, revealed blooms throughout the first three years of monitoring, except for 2023, when concentrations decreased notably (Fig. 2), reaching the maximum value of 11.44 µg.L^−1^, as opposed to 240.22 µg.L^−1^ in the beginning of the monitoring period (2020). The production of biomass with the availability of inorganic nutrients is shown by the direct correlation of chlorophyll-*a* with NH_4_^+^ and DIP. The small decrease in the concentrations of DIP and DIN throughout the monitoring period could relate to the decrease of the phytoplankton blooms in the same period.

The decrease of DIP from 2020 to 2023, despite initially elevated concentrations in fluvial waters (up to 142.92 µM), suggests a progressive shift in nutrient dynamics within the drainage basin. In eutrophic aquatic systems, phosphorus availability is controlled not only by external loading but also by internal cycling processes, particularly organic matter mineralization and sediment–water exchange (Boström et al., 1988; Golterman, 2001; Søndergaard et al., 2013). Both DIP and inorganic nitrogen forms can be regenerated through microbial decomposition of organic matter, a process that intensifies under warm and oxygen depleted conditions, typical of tropical eutrophic systems. This is consistent with the significant inverse correlations between NH₄⁺ and dissolved oxygen, and between DIP and dissolved oxygen, indicating oxygen consumption during organic matter degradation and simultaneous release of inorganic nutrients to the water column. Such coupling between oxygen depletion and nutrient regeneration is a well-established mechanism in eutrophic and organically enriched aquatic environments (Edwards et al., 2024; Viaroli et al., 2008).

High turbidity values observed in the system, reaching up to 962 NTU, further indicate substantial particulate and organic matter inputs, which may originate from multiple anthropogenic sources, including untreated wastewater, urban runoff, and resuspension of bottom sediments. In highly urbanized catchments, these inputs are typically episodic but recurrent, contributing to sustained nutrient and organic matter loading rather than isolated events. Although sewage inputs constitute a relevant external source of nutrients, the interpretation of system dynamics cannot be reduced solely to wastewater discharge. Instead, nutrient availability reflects the interaction between external loading and internal regeneration processes, which together regulate phytoplankton biomass and dissolved nutrient concentrations (Pereira & Mulligan, 2023). The observed correlation between DO and DIP supports this coupling between nutrient availability and consumption of dissolved oxygen during OM degradation. The decline in phytoplankton biomass observed in 2023, despite continued presence of nutrients, may reflect shifts in light limitation associated with turbidity reduction, changes in residence time, or altered nutrient stoichiometry affecting phytoplankton growth efficiency.

The application of effective microorganisms (EM) is intended to enhance organic matter mineralization by stimulating microbial activity, thereby accelerating the transformation of particulate and dissolved organic material into inorganic nutrient forms. This mechanism depends strongly on substrate availability, particularly organic carbon inputs, which regulate microbial metabolism and nutrient recycling rates in aquatic systems (Lewicka-Rataj et al., 2024). The literature regarding the effectiveness of EM technology is still scarce, with different applications that describe notable improvement of water quality for sewage treatment as described in the study of (Monica et al., 2011), and in the restoration of a water body as reported in the study of (Sitarek et al., 2017). Other studies have described the increase in organic matter mineralization and increase in phytoplankton abundance on the one hand (Dondajewska et al., 2019), and the failure in the reduction of nitrogen and phosphorus contents on the other hand (Dondajewska et al., 2019; Lürling et al., 2016). Apart from nutrient load reduction and acceleration of organic matter mineralization, some authors (Araújo et al., 2016; Liu et al., 2018) consider water transparency and total suspended solids as the main criteria for evaluation of water quality. Positive effects arising from the use of EM technology in polluted reservoir in Poland were registered by Mazurkiewicz et al. (2020), who reported not only the increase of transparency, but also reduction of nitrogen and phosphorus loads as well as decrease of sedimentary organic matter and putrefactive odors.

The waters of the Mumbuca-Ubatiba drainage basin started to be treated with EM periodically at the end of 2021. Since then, some positive changes have been registered, such as the small decreases in DIN, PO_4_^3−^, turbidity, phytoplankton biomass, and a slight increase in dissolved oxygen levels. Despite that, the occurrence of hypoxia events continued to be registered, as well as high loads of nitrogen and phosphorus, obliterating the effects of bioremediation, since this mitigation measure was not properly accompanied by sewage treatment in the urbanized areas. The presence of excess aquatic weeds might also be a side effect of bioremediation with EM, loading the waters with floating mats as a side effect from inorganic forms of nutrients that arise from organic matter decomposition.

On the west side of Maricá lagoon São Bento channel constitutes a significant source of anthropogenic loads (Fig. 1). Hypoxia persisted throughout the monitoring period in this water course, with DO concentrations below 2.0 mg.L^−1^ detected in almost every campaign, with a higher frequency in dry periods. The regular input of nitrogen forms in São Bento channel might have multiple sources such as domestic effluents and fertilizers used in agriculture, reflecting the changes in coverage and use of soils in the surroundings of Maricá lagoon in the past decades as show in the study of Amora-Nogueira et al. (2023) that unveiled eutrophication in Maricá lagoon following deforestation, agricultural development, urban growth and lack of seawage treatment along the last 120 years. Regarding DIN in São Bento channel, nitrate was the dominant form throughout most of the monitored period, composing > 80% of nitrogen pool, and contrasting with the results found in the Mumbuca-Ubatiba drainage basin, where ammonium was the dominant nitrogen form. The onlrophylly period when ammonium concentrations predominated in the composition of DIN was August/2023, suggesting degradation of organic matter, which was corroborated by the low levels of DO and elevated concentrations of inorganic nitrogen. Chlorophyll-*a* presented concentrations that characterized phytoplankton blooms in some campaigns and revealed effects of seasonality with significant differences between dry and wet seasons. Total phosphorus concentrations also varied seasonally, probably a result of higher primary production in wet season. Another remarkable difference between the waters of São Bento Channel and Mumbuca-Ubatiba drainage basin was turbidity. Despite presenting elevated turbidity waters, the values registered in São Bento Channel were much lower, suggesting a lower anthropogenic load in the west margin of the lagoon, and might as well represent the effect of marine waters received in this part of the lagoon.

The marked differences in nutrients concentrations between São Bento channel and Mumbuca-Ubatiba drainage basin stood out before the beginning of bioremediation in the latter, reflecting not only the differences in the degree of urbanization and size of the drained area on opposite margins of the lagoon, but also the economic activities revealed by the different forms of nitrogen. The agropasture area in the drainage basin of Mumbuca-Ubatiba is bigger, corroborating the hypotheses of a great load of organic matter in the east margin of the lagoon. There is also an area with higher population density close to the discharge of Mumbuca river. On the west margin, the agropasture area is smaller and there is a larger area of human occupation. The north portion of the lagoon includes a small area with population density and a larger one with rarefied human occupation (Ferreira, 2017).

Results of Mumbuca-Ubatiba suggest massive discharge of organic matter, resulting in high levels of ammonium after mineralization, especially considering the larger urbanized area that is drained. On the other hand, contribution of nitrogen forms to the lagoon through São Bento channel arrives mostly in the form of nitrate, which might have different anthropogenic origins varying from runoff of fertilized lawns and cropland, animal manure storage areas and landfills to atmospheric deposition. Differences in concentrations and nitrogen forms, especially ammonium and nitrate, also stood out between the main fluvial sources in the lagoon. The Mumbuca-Ubatiba drainage basin has also revealed larger input of dissolved inorganic phosphorus, since concentrations of DIP were of the same magnitude as total dissolved phosphorus (TDP) registered in São Bento Channel.

In spite of the high loads of inorganic nutrients that reach the waters of Maricá lagoon through Mumbuca-Ubatiba rivers and São Bento channel, concentrations are much lower than the values registered in the fluvial sources. The health status of Maricá lagoon has been monitored in the past decades by public authorities (Ferreira, 2017) and variations of DIP, NO_2_^−^, NO_3_^−^ and NH_4_^+^, between 2010 and 2014, were respectively: 0.83–11.45, 0.02–26.08, 0.16–1.93, and 0.56–6.67 µM, much lower than the ones registered in the present study, unveiling the increase of anthropogenic impacts in this ecosystem since then.

The increase in inorganic dissolved nutrients in the Mumbuca-Ubatiba drainage basin over the past decades was unequivocally demonstrated by comparison with the study of Ferreira (2017), which reported much lower concentrations of DIP, NO₂⁻, NO₃⁻, and NH₄⁺. The values were 3.12 to 67.67 µM for DIP, 3.69 to 7.82 µM for NO₂⁻, 9.4 to 111.10 µM for NO₃⁻, and 1.77 to 5.48 µM for NH₄⁺, respectively, much lower when compared to current levels.

Nutrients entering lagoon systems are used in primary production, and excess autochthonous production is likely to accumulate in the muddy bottom of the system, a process favored by low depth (Domingues, 2022; Pérez-Martín, 2023). Regarding the signs that might indicate positive effects of bioremediation in the lagoon, the rise in dissolved oxygen levels throughout the sampling period suggested the improvement of the ecosystem health, as shown by the increase of the lower quartiles after the beginning of EM use in the drainage basin. Other visible signs of the improvement of water quality are the decrease in turbidity and TDP concentrations between the first and last campaigns. The decrease of DIN concentrations was not so noticeable as expected, due to the large inputs of nitrogen on the east and west margins of the lagoon. Although DIN concentrations increased during the August 2023 survey, this variation likely reflects the high temporal variability of dissolved nutrients in the water column and cannot be unequivocally attributed to changes in EM effectiveness. No specific environmental disturbance was identified during the study period, and unmonitored variations in external nutrient inputs may also have contributed to the observed pattern.

Whereas water quality alone may not be a robust tool to evaluate ecosystem health because of faster dynamics and diurnal variations (Huang et al., 2025), sedimentary results act as a geological record (Sun et al., 2021). The decrease in TOC concentrations post bioremediation has been the most remarkable change throughout this monitoring. Total organic carbon reached maximum concentrations of 7.79 and 2.72% in 2021 and 2023, respectively. The concentrations of TOC in Maricá lagoon were much higher than the values registered in 2013 (Laut et al., 2019) that varied between 0.2 and 3.8%, showing the increase of anthropogenic input in the ecosystem in the past decades. The decrease of TOC concentrations to levels closer to the ones found over a decade ago are a good indicator of the positive effects of bioremediation. Levels of TOC before bioremediation were quite similar, or even higher to other coastal lagoons impacted by anthropogenic activities such as Rodrigo de Freitas lagoon, with TOC concentrations between 0.3 and 6.7% (Vezzone et al., 2021), Tijuca Lagoon with TOC varying between 1.9 and 4.9% (Teixeira et al., 2023) and Xincun Bay with values between 0.17 and 3.27% (Hao et al., 2024).

Total phosphorus presented a trend to decrease between February/22 and May/23, suggesting the recycling of phosphorus rather than accumulation in bottom sediments. In August/23 an expressive increase in TP concentration was registered, reaching the maximum value during the monitoring period, 2826 mg.kg^−1^. Stations P16 and P20 to P29 (Fig. 1C) presented a marked increase in TP concentrations in this period (1113–2826 mg.kg^−1^). Stations P20–P29 are mainly in the area under the influence of Mumbuca-Ubatiba drainage basin and might represent an isolated event, through increase of the anthropogenic load. Phytoplankton biomass accompanied this TP increase in the same month, suggesting an input of excess phosphorus between May and August/2023. Continuing the monitoring would be necessary to evaluate this hypothesis, since the observed trend was the decrease of TOC and TP along the monitored period. The levels of TP found in Maricá lagoon were similar to the ones found in Itaipu lagoon (60–1356 mg.kg^−1^) (Lobo et al., 2023), a eutrophic coastal system in the state of Rio de Janeiro.

### Trophic index for Maricá Lagoon

**Fig. 6:**
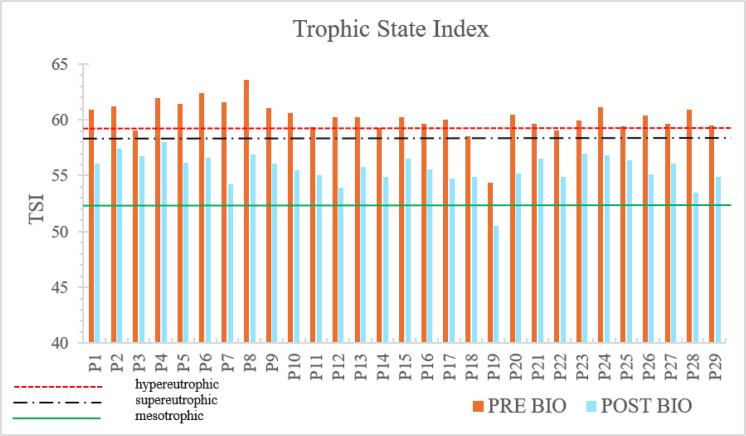
Mean trophic state index (TSI) for Maricá waters before (PREBIO) and after (POST BIO) bioremediation with EM®

The trophic state index (TSI) revealed a general reduction in the trophic state of the Maricá lagoon, shifting from eutrophic to mesotrophic conditions (Fig. 6). Prior to bioremediation, all sampling stations were classified between hypereutrophic (TSI ≥ 59.1) and supertrophic (58.2 ≤ TSI ≤ 59). The stations with the highest trophic levels were predominantly located within the influence area of the Mumbuca-Ubatiba drainage basin (P1–P10, P20, P24, and P26). Additionally, stations P17, P28, and P29, which also exhibited elevated TSI values, were closer to the influence of the São Bento Channel.

Following bioremediation, many stations remained within the supereutrophic range; however, a noticeable improvement was observed in several sites, which shifted to mesotrophic conditions (53.2 ≤ TSI ≤ 55.7). This reduction in trophic state was recorded both in stations directly influenced by the Mumbuca River (e.g., P7, P20, P22, and P26) and in stations located farther from its direct influence (e.g., P11, P12, P14, P17, P18, P28, and P29), indicating a broader spatial impact of the bioremediation efforts.

## Limitations of the study

Despite providing a long-term data set, the study has limitations considering the complexity of lagoon systems. Several studies regarding bioremediation using EM are usually conducted on smaller scales and controlled conditions in the laboratory, whereas the present study was made in a natural environment subjected to strong seasonal variations and continuous anthropogenic pressure, which may have influenced the outcomes.

Coastal lagoons are highly dynamic systems, and biogeochemical processes such as nutrient cycling, sediment interactions with water column, and biological responses can occur over longer timescales than the ones covered in this study.

Finally, the study focused primarily on physicochemical and biogeochemical variables with limited assessment of biological communities and microbial dynamics. A broader evaluation, including microbial community structure and ecosystem-level responses, as well as investigation of sedimentary biogeochemical processes would provide a deeper understanding of the mechanisms driving the observed changes and the overall effectiveness of EM-based bioremediation.

Another limitation of the present study is that the ecological fate of effective microorganisms after their introduction into the lagoon system was not assessed. Future studies should investigate the persistence of introduced microbial populations, their interactions with native microbial communities, their incorporation into the microbial food web, and any potential long-term ecological consequences associated with repeated EM applications.

Future investigations would benefit from the inclusion of untreated reference sites or comparable coastal lagoons, together with an expanded spatial sampling design and a broader suite of physicochemical and biogeochemical analyses. The implementation of such an approach will depend on the availability of additional financial and logistical resources, given the high costs associated with field campaigns and laboratory analyses in long-term monitoring programs.

## Conclusion

This study achieved its main objective of evaluating the effects of anthropogenic nutrient inputs and EM-based bioremediation on the water quality and trophic status of the Maricá Lagoon system. The results demonstrate that the Mumbuca-Ubatiba drainage basin is a major source of nutrient enrichment, particularly ammonium, probably associated with the decomposition of organic matter derived from untreated sewage inputs, which contributes to seasonal hypoxic conditions and strong ecosystem variability.

The monitoring also showed distinct nutrient signatures between the two main tributaries, with nitrate predominating in the São Bento Channel and ammonium in the Mumbuca-Ubatiba basin, highlighting spatial heterogeneity in nitrogen sources and transformations within the watershed.

The observed improvements in water quality, trophic status, and sediment characteristics suggest that the application of effective microorganisms may contribute to the recovery of degraded lagoon ecosystems when implemented as a nature-based solution. Although the present study does not establish a direct causal relationship between EM application and all observed environmental changes, the temporal patterns identified are consistent with a positive ecological response following the intervention.

These findings suggest that EM-based bioremediation represents a promising environmental management approach but requires further evaluation through long-term monitoring and controlled experimental studies. However, for such interventions to achieve long-lasting environmental benefits, they should be integrated with broader watershed management measures, particularly the implementation of adequate sewage treatment infrastructure. The monitoring conducted in this study proved valuable for assessing ecosystem responses over time and provides useful information to support management decisions and public policies for anthropogenically impacted coastal lagoons.

## Supplementary Information

Below is the link to the electronic supplementary material.

ESM 1 (DOCX 41.7 KB)

## Data Availability

The dataset will be made available under demand.

## Declarations

All authors have read, understood, and have complied as applicable with the statement on “Ethical responsibilities of Authors” as found in the Instructions for Authors.
