# Supplementary material for: Monitoring bioremediation of an impacted coastal lagoon through nature-based solutions: a case study of the Maricá Lagoon (RJ, Brazil)
Source: Environ Monit Assess. 2026 Jul 24;198(8):874. doi: 10.1007/s10661-026-15699-y (PMC13400587; doi:10.1007/s10661-026-15699-y)
Supplement: Supplementary file 1 — (DOCX 41.7 KB) [file 10661_2026_15699_MOESM1_ESM.docx]

**Appendix A: Supplementary material**

Table S1: Results of Mann-Whitney test (*p<0.050*) including effect size (r) for temperature, salinity, pH, dissolved oxygen, turbidity, dissolved oxygen (DO), dissolved inorganic phosphate (DIP), nitrite, nitrate, ammonium, dissolved inorganic nitrogen (DIN) in Mumbuca-Ubatiba drainage basin between dry and wet seasons.

| **Variables** | **U** | **Z** | ***p*** | **r** |
| --- | --- | --- | --- | --- |
| Temperature | 3332.50 | -12.3617 | 0.000000 | 0.7 |
| Salinity | 10441.50 | 4.6102 | 0.000004 | 0.2 |
| pH | 11333.00 | 3.6381 | 0.000275 | 0.2 |
| DO | 12350.00 | -2.5292 | 0.011434 | 0.1 |
| Turbidity | 11146.00 | 1.9036 | 0.056968 | 0.1 |
| Chlorophyll-*a* | 9923.00 | 2.4853 | 0.012946 | 0.1 |
| DIP^-^ | 9844.00 | 4.3192 | 0.000016 | 0.2 |
| NO_2_^-^ | 10371.50 | 1.7683 | 0.077020 | 0.1 |
| NO_3_^-^ | 10140.00 | 0.2198 | 0.826040 | 0.0 |
| NH_4_^+^ | 10330.00 | 3.9415 | 0.000081 | 0.2 |
| DIN | 10020.00 | 4.3646 | 0.000013 | 0.2 |

Table S2: Results of Mann-Whitney test (*p<0.050*) including effect size (r) for temperature, salinity, pH, dissolved oxygen, turbidity, dissolved oxygen (DO), dissolved inorganic phosphate (DIP), nitrite, nitrate, ammonium, total dissolved nitrogen (DIN) in Mumbuca-Ubatiba drainage basin between pre and post bioremediation in wet season.

| **Variables** | **U** | **Z** | ***p*** | **r** |
| --- | --- | --- | --- | --- |
| Temperature | 2267.500 | -4.04193 | 0.000053 | 0.3 |
| Salinity | 2965.000 | -1.92538 | 0.054183 | 0.1 |
| pH | 3265.000 | 1.01504 | 0.310090 | 0.1 |
| DO | 3522.500 | 0.23366 | 0.815253 | 0.0 |
| Turbidity | 1613.500 | -4.61172 | 0.000004 | 0.4 |
| Chlorophyll-*a* | 2437.500 | 3.52607 | 0.000422 | 0.3 |
| DIP | 3064.000 | 1.62497 | 0.104171 | 0.1 |
| NO_2_^-^ | 2340.000 | -2.04472 | 0.040883 | 0.2 |
| NO_3_^-^ | 1720.000 | -1.25853 | 0.208200 | 0.1 |
| NH_4_^+^ | 2116.000 | 4.44140 | 0.000009 | 0.3 |
| DIN | 2131.000 | 4.45614 | 0.000008 | 0.3 |

Table S3: Results of Mann-Whitney test (*p<0.050*) including effect size (r) for temperature, salinity, pH, dissolved oxygen, turbidity, dissolved oxygen (DO), dissolved inorganic phosphate, nitrite, nitrate, ammonium, total dissolved nitrogen (DIN) in Mumbuca-Ubatiba drainage basin between pre and post bioremediation in dry season.

| **Variables** | **U** | **Z** | ***p*** | **r** |
| --- | --- | --- | --- | --- |
| Temperature | 2437.000 | 2.80211 | 0.005077 | 0.2 |
| Salinity | 3075.000 | 0.67003 | 0.502837 | 0.1 |
| pH | 2276.500 | 3.33847 | 0.000843 | 0.3 |
| DO | 2337.500 | -3.13462 | 0.001721 | 0.2 |
| Turbidity | 1842.000 | -4.79048 | 0.000002 | 0.4 |
| Chlorophyll-*a* | 2095.500 | 0.29248 | 0.769916 | 0.0 |
| DIP | 1317.000 | 5.62688 | 0.000000 | 0.5 |
| NO_2_^-^ | 1510.000 | 4.41599 | 0.000010 | 0.4 |
| NO_3_^-^ | 1668.000 | 3.47450 | 0.000512 | 0.3 |
| NH_4_^+^ | 2709.000 | 0.87814 | 0.379869 | 0.1 |
| DIN | 2586.000 | 1.32355 | 0.185655 | 0.1 |

Table S4: Spearman analysis for for temperature, salinity, pH, dissolved oxygen, turbidity, dissolved oxygen, dissolved inorganic phosphate, dissolved nitrite, nitrate, ammonium, dissolved nitrogen (DIN) in Mumbuca-Ubatiba drainage basin.

|  | T | pH | S | DO | turbidity | Chlorophyll-a | DIN | NH_4_^+^ | NO_3_^-^ | NO_2_^-^ | DIP |
| --- | --- | --- | --- | --- | --- | --- | --- | --- | --- | --- | --- |
| T | 1.00 | -0.07 | -0.06 | 0.01 | -0.01 | 0.10 | -0.04 | -0.02 | **-0.12** | **-0.14** | -0.07 |
| pH |  | 1.00 | 0.10 | 0.01 | -0.03 | 0.06 | 0.09 | 0.09 | 0.08 | **0.11** | **0.12** |
| S |  |  | 1.00 | **-0.30** | 0.08 | **0.29** | **0.48** | **0.48** | **-0.50** | **-0.36** | **0.40** |
| DO |  |  |  | 1.00 | -0.04 | 0.00 | **-0.45** | **-0.45** | **0.35** | **0.33** | **-0.42** |
|  |  |  |  |  |  |  |  |  |  |  |  |
| turbidity |  |  |  |  | 1.00 | 0.07 | **0.14** | **0.14** | **-0.14** | **-0.17** | 0.06 |
| Chlorophyll-a |  |  |  |  |  | 1.00 | **0.16** | **0.17** | **-0.32** | **-0.20** | **0.14** |
| DIN |  |  |  |  |  |  | 1.00 | **1.00** | **-0.45** | **-0.55** | **0.74** |
| NH_4_^+^ |  |  |  |  |  |  |  | 1.00 | **-0.49** | **-0.59** | **0.73** |
| NO_3_^-^ |  |  |  |  |  |  |  |  | 1.00 | **0.68** | **-0.29** |
| NO_2_^-^ |  |  |  |  |  |  |  |  |  | 1.00 | **-0.34** |
| DIP |  |  |  |  |  |  |  |  |  |  | 1.00 |

Table S5: Results of Mann-Whitney test (*p<0.050*) including effect size (r) for temperature, salinity, pH, dissolved oxygen (DO), turbidity, chlorophyll-*a,* total dissolved phosphorus (TDP), ammonium, dissolved nitrite, nitrate, ammonium, dissolved inorganic nitrogen (DIN) in São Bento channel between dry and wet seasons.

| **Variables** | **U** | **Z** | ***p*** | **r** |
| --- | --- | --- | --- | --- |
| Temperature | 0.0000 | 6.13527 | 0.000000 | 0.9 |
| Salinity | 113.0000 | -4.77425 | 0.000002 | 0.6 |
| pH | 404.5000 | 0.24105 | 0.809520 | 0.0 |
| DO | 393.5000 | 0.41211 | 0.680260 | 0.1 |
| Turbidity | 125.5000 | 4.57986 | 0.000005 | 0.6 |
| Chlorophyll-*a* | 81.0000 | -3.61586 | 0.000299 | 0.6 |
| TDP | 239.0000 | -2.81478 | 0.004881 | 0.4 |
| NO_2_^-^ | 19.0000 | -1.58792 | 0.112305 | 0.3 |
| NO_3_^-^ | 321.0000 | 1.53958 | 0.123664 | 0.2 |
| NH_4_^+^ | 371.5000 | -0.75424 | 0.450707 | 0.1 |
| DIN | 382.5000 | -0.99056 | 0.321903 | 0.1 |

Table S6: Results of Mann-Whitney test (*p<0.050*) including effect size (r) for temperature, salinity, pH, dissolved oxygen (DO), turbidity, chlorophyll-*a,* total dissolved phosphorus (TDP), ammonium, dissolved nitrite, nitrate, ammonium, dissolved inorganic nitrogen (DIN), total sedimentary phosphorus (TP) and total organic carbon (TOC) in Maricá lagoon between dry and wet seasons.

| **Variables** | **U** | **Z** | ***p*** | **r** |
| --- | --- | --- | --- | --- |
| Temperature | 195.000 | 13.55527 | 0.000000 | 0.8 |
| Salinity | 5576.500 | -6.91206 | 0.000000 | 0.4 |
| pH | 7489.500 | 4.23294 | 0.000023 | 0.2 |
| DO | 6719.500 | -5.31131 | 0.000000 | 0.3 |
| Turbidity | 9153.000 | -1.90325 | 0.057009 | 0.1 |
| Chlorophyll-*a* | 3375.000 | -5.50761 | 0.000000 | 0.4 |
| TDP | 4562.500 | 8.27336 | 0.000000 | 0.5 |
| NO_2_^-^ | 5629.500 | -6.83783 | 0.000000 | 0.4 |
| NO_3_^-^ | 5895.000 | -6.46600 | 0.000000 | 0.4 |
| NH_4_^+^ | 9015.500 | 2.09581 | 0.036099 | 0.1 |
| DIN | 9015.500 | 2.09581 | 0.036099 | 0.1 |
| TP | 6937.000 | -4.85441 | 0.000001 | 0.3 |
| TOC | 8336.500 | -0.12046 | 0.904117 | 0.0 |

Table S7: Results of Mann-Whitney test (*p<0.050*) including effect size (r) for temperature, salinity, pH, dissolved oxygen (DO), turbidity, chlorophyll-*a,* total dissolved phosphorus (TDP), ammonium, dissolved nitrite, nitrate, ammonium, dissolved inorganic nitrogen (DIN), total sedimentary phosphorus (TP) and total organic carbon (TOC) in Maricá lagoon between pre and post bioremediation for dry season.

| **Variables** | **U** | **Z** | ***p*** | **r** |
| --- | --- | --- | --- | --- |
| pH | 1494.500 | 0.92433 | 0.355314 | 0.1 |
| DO | 835.500 | -4.18174 | 0.000029 | 0.3 |
| Turbidity | 223.500 | 7.20682 | 0.000000 | 0.6 |
| chlo-a | 941.500 | 1.41068 | 0.158339 | 0.1 |
| TDP | 161.000 | 7.50211 | 0.000000 | 0.6 |
| NO_2_^-^ | 1169.000 | -2.53326 | 0.011301 | 0.2 |
| NO_3_^-^ | 1222.000 | 2.27129 | 0.023130 | 0.2 |
| NH_4_^+^ | 1076.000 | 2.99296 | 0.002763 | 0.2 |
| TDN | 1126.000 | 2.74581 | 0.006037 | 0.2 |
| TP | 676.000 | 4.93665 | 0.000001 | 0.4 |
| TOC | 386.000 | 5.57888 | 0.000000 | 0.5 |

Table S8: Results of Mann-Whitney test (*p<0.050*) including effect size (r) for temperature, salinity, pH, dissolved oxygen (DO), turbidity, chlorophyll-*a,* total dissolved phosphorus (TDP), ammonium, dissolved nitrite, nitrate, ammonium, dissolved inorganic nitrogen (DIN), total sedimentary phosphorus (TP) and total organic carbon (TOC) in Maricá lagoon between pre and post bioremediation for wet season.

| **Variables** | **U** | **Z** | ***p*** | **r** |
| --- | --- | --- | --- | --- |
| pH | 1634.000 | -0.23479 | 0.814372 | 0.0 |
| DO | 1032.500 | -3.20797 | 0.001337 | 0.3 |
| Turbidity | 1351.500 | -1.63117 | 0.102855 | 0.1 |
| chlo-a | 0.000 | 0.00000 | 1.000000 | 0.0 |
| TDP | 1.000 | 8.30663 | 0.000000 | 0.7 |
| NO_2_^-^ | 1135.000 | 2.70132 | 0.006907 | 0.2 |
| NO_3_^-^ | 1161.000 | 2.57281 | 0.010088 | 0.2 |
| NH_4_^+^ | 1251.000 | -2.12794 | 0.033343 | 0.2 |
| TDN | 1406.000 | 1.36178 | 0.173267 | 0.1 |
| TP | 0.000 | -8.30413 | 0.000000 | 0.7 |
| TOC | 960.500 | 3.56387 | 0.000365 | 0.3 |
